# Difamilast, a Topical Phosphodiesterase 4 Inhibitor, Produces Soluble ST2 via the AHR–NRF2 Axis in Human Keratinocytes

**DOI:** 10.3390/ijms25147910

**Published:** 2024-07-19

**Authors:** Gaku Tsuji, Ayako Yumine, Koji Kawamura, Masaki Takemura, Makiko Kido-Nakahara, Kazuhiko Yamamura, Takeshi Nakahara

**Affiliations:** 1 Research and Clinical Center for Yusho and Dioxin, Kyushu University Hospital, Fukuoka 812-8582, Japan; yumine.ayako.977@m.kyushu-u.ac.jp (A.Y.); yamamura.kazuhiko.821@m.kyushu-u.ac.jp (K.Y.); nakahara.takeshi.930@m.kyushu-u.ac.jp (T.N.); 2 Department of Dermatology, Graduate School of Medical Sciences, Kyushu University, Fukuoka 812-8582, Japan; kawamura.koji.570@s.kyushu-u.ac.jp (K.K.); takemura.masaki.618@m.kyushu-u.ac.jp (M.T.); nakahara.makiko.107@m.kyushu-u.ac.jp (M.K.-N.)

**Keywords:** phosphodiesterase 4, IL-33, soluble ST2, aryl hydrocarbon receptor, nuclear factor erythroid 2-related factor 2, atopic dermatitis

## Abstract

Difamilast, a phosphodiesterase 4 (PDE4) inhibitor, has been shown to be effective in the treatment of atopic dermatitis (AD), although the mechanism involved remains unclear. Since IL-33 plays an important role in the pathogenesis of AD, we investigated the effect of difamilast on IL-33 activity. Since an in vitro model of cultured normal human epidermal keratinocytes (NHEKs) has been utilized to evaluate the pharmacological potential of adjunctive treatment of AD, we treated NHEKs with difamilast and analyzed the expression of the suppression of tumorigenicity 2 protein (ST2), an IL-33 receptor with transmembrane (ST2L) and soluble (sST2) isoforms. Difamilast treatment increased mRNA and protein levels of sST2, a decoy receptor suppressing IL-33 signal transduction, without affecting ST2L expression. Furthermore, supernatants from difamilast-treated NHEKs inhibited IL-33-induced upregulation of TNF-α, IL-5, and IL-13 in KU812 cells, a basophil cell line sensitive to IL-33. We also found that difamilast activated the aryl hydrocarbon receptor (AHR)–nuclear factor erythroid 2-related factor 2 (NRF2) axis. Additionally, the knockdown of AHR or NRF2 abolished the difamilast-induced sST2 production. These results indicate that difamilast treatment produces sST2 via the AHR–NRF2 axis, contributing to improving AD symptoms by inhibiting IL-33 activity.

## 1. Introduction

Atopic dermatitis (AD) is a chronic relapsing inflammatory skin disease characterized by dry skin, eczematous lesions, and pruritus [1]. Patients with AD also complain of a burden of sleep disturbance leading to anxiety, depression, and decreased quality of life [2]. Despite the efficacy of topical corticosteroids as an initial treatment for AD patients, their long-term use is associated with side effects and safety concerns, leading to poor adherence [3]. Therefore, the development of a new topical agent for AD is desirable.

Although the pathogenesis of AD is multifactorial, it mainly involves the interaction of three factors: type 2 immune response, skin barrier dysfunction, and pruritus [4]. Recently, the role of phosphodiesterase 4 (PDE4), an enzyme that degrades intracellular cyclic adenosine monophosphate (cAMP), in the pathogenesis of AD has been demonstrated [5]. PDE4 is expressed in human keratinocytes and Langerhans cells [6]. Increased PDE4 activity and decreased intracellular cAMP levels are reported to lead to the production of inflammatory mediators [6]. Recently, PDE4 inhibition has been reported to decrease IL-4 production by basophils in an AD mouse model [7].

Difamilast, a topical PDE4 inhibitor, is now available in Japan for the treatment of adult and pediatric AD patients (aged ≥ 3 months). Phase III studies have shown a favorable efficacy and safety profile of difamilast treatment in AD [8], but the mechanism involved is still unclear. We previously showed that difamilast treatment increases the expression of filaggrin and loricrin, important molecules for skin barrier function, in human keratinocytes [5].

IL-33 is an IL-1 family cytokine reported to be expressed on keratinocytes, fibroblasts, vascular endothelial cells, dendritic cells, macrophages, and mast cells [9]. IL-33 is localized to the nucleus and is secreted following cell death, such as necrosis induced by scratching [10] and necroptosis induced by streptococcal infection [11]. Two types of isoforms of the IL-33 receptor, suppression of tumorigenicity 2 protein (ST2), have been identified: transmembrane ST2L and soluble ST2 (sST2) [12]. Binding of IL-33 to ST2L induces MyD88-dependent activation of nuclear factor-kappa B (NF-κB) and mitogen-activated protein kinase (MAPK) as intracellular signaling [12]. Since sST2 lacks an intracellular signaling domain, it is thought to be a decoy receptor to suppress inflammatory signaling induced by IL-33 [12]. Several studies have reported that the administration of sST2 suppresses IL-33-induced activation in mast cells [13] and group 2 innate lymphoid cells (ILC2s) [14] and airway inflammation in a murine asthma model [15,16].

It has also been reported that repeated injection of IL-33 or overexpression of IL-33 in the skin leads to the development of AD-like skin inflammation in a mouse model [17]. In addition, mice lacking IL-33 in the epidermis did not develop severe AD-like skin lesions induced by MC903, a vitamin D analog [17]. The response of IL-33 to immune cells includes the stimulation of mast cells and ILC2s, which contribute to the production of type 2 cytokines such as IL-5 and IL-13 [17,18]. Th2 cells are also stimulated by IL-33, resulting in the production of IL-4 and IL-13 [17,18]. In addition, IL-33 decreases the expression of filaggrin and loricrin, contributing to skin barrier dysfunction [17,18]. Furthermore, IL-33 sensitizes sensory nerves to cause intense pruritus, which contributes to scratching behavior [19]. IL-33 is also reported to be highly expressed in the nuclei of keratinocytes in human AD lesions [20]. These findings suggest that IL-33 plays an important role in the pathogenesis of AD and that the inhibition of IL-33 activity will be beneficial for the treatment of AD. Against this background, we hypothesized that difamilast treatment could modulate IL-33 activity in AD.

Cultured normal human epidermal keratinocytes (NHEKs) have been utilized to evaluate the pharmacological potential of new active ingredients for adjunctive treatment of AD employing in vitro models [21]. Keratinocytes, a major component of the epidermis, play an important role in polarizing the immune response towards the Th2 allergic response, such as in AD [21]. In addition, the use of a single cell type allows evaluation of the specific keratinocyte response. In situations where AD is becoming milder, since lesions and normal skin are interspersed with each other, and it is difficult to clearly distinguish the area of topical application, the topical site of difamilast includes the normal skin. Therefore, it is important to analyze the effects of difamilast on normal skin in order to obtain a better understanding of the pathogenesis and treatment of AD. Based on this background, we analyzed the expression of IL-33, ST2L, and sST2 in NHEKs treated with difamilast.

## 2. Results

### 2.1. Difamilast Treatment Produced sST2 in NHEKs

**Figure 1:**
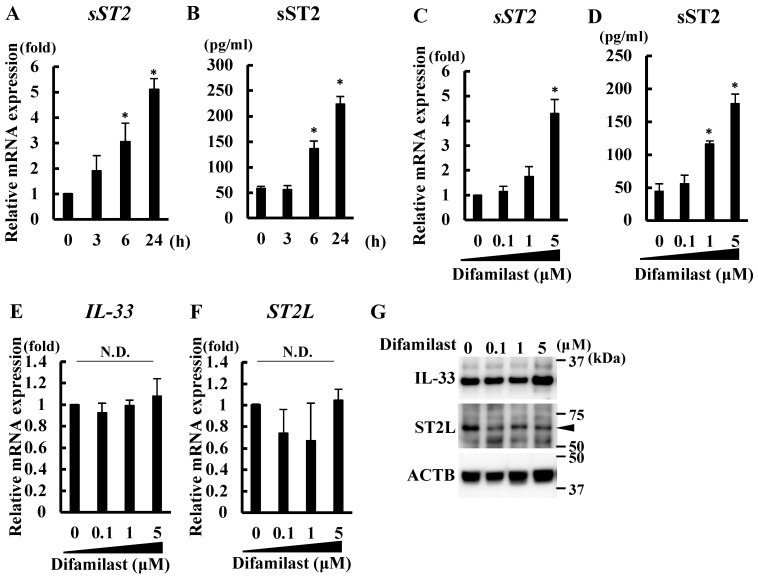
Difamilast treatment upregulated sST2 in NHEKs. (**A**,**B**) NHEKs were treated with difamilast (5 μM) for 3, 6, or 24 h. (**C**–**G**) NHEKs were treated with 0.1, 1, or 5 μM difamilast for 24 h. (**A**,**C**,**E**,**F**) qRT-PCR. (**B**,**D**) ELISA on supernatant. (**G**) Western blotting. (**A**–**F**) Data are expressed as mean ± S.D.; N = 3/group. * Significant difference between difamilast-treated group and control group (*p* < 0.05); Dunnett’s multiple comparison test; N.D.: No significant difference. (**G**) Representative images from triplicate experiments with similar results are shown.

We previously showed that difamilast treatment (1 or 5 μM) induces an increase in intracellular cAMP levels and phosphorylation of cAMP response element binding protein (CREB) without cell toxicity [5]. In this study, we treated NHEKs with difamilast (5 μM) for 3, 6, or 24 h and found that such treatment increased sST2 mRNA and protein levels at 6 and 24 h (Figure 1A,B). We also confirmed that difamilast treatment increased sST2 protein level at 1 μM and sST2 mRNA and protein levels at 5 μM (Figure 1C,D). In addition, we examined the mRNA and protein levels of IL-33 and ST2L. We did not observe any significant changes in IL-33 and ST2L expression in NHEKs treated with difamilast (0.1, 1, and 5 μM) for 24 h (Figure 1E–G). These results indicate that difamilast treatment upregulates sST2 without affecting IL-33 and ST2L expression in NHEKs.

### 2.2. Difamilast Treatment Inhibited IL-33 Activity via sST2 Production by NHEKs

**Figure 2:**
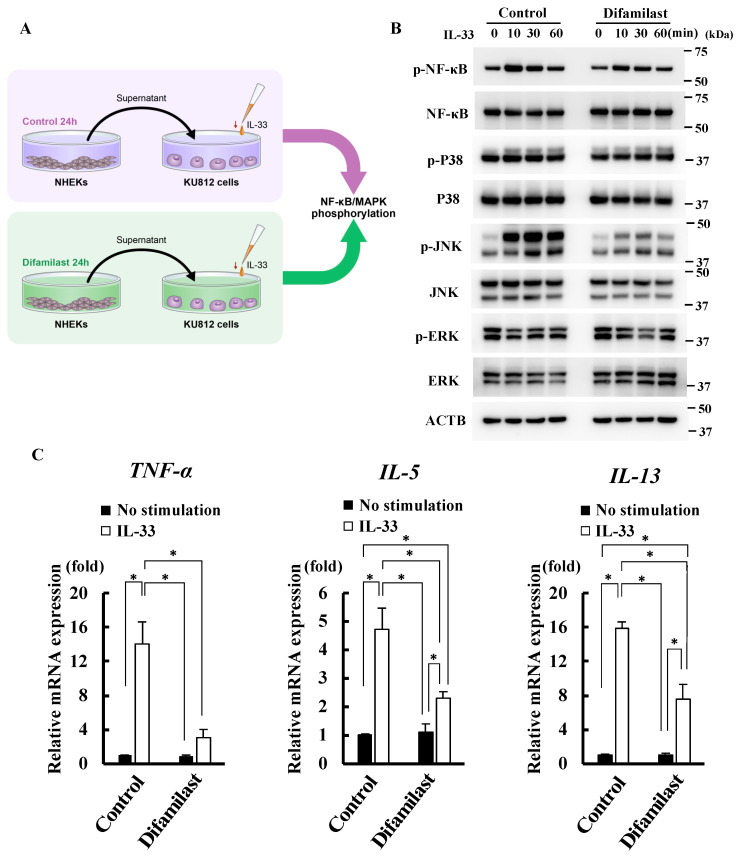
Difamilast treatment may inhibit the activity of IL-33 via sST2 production by NHEKs. (**A**) A schematic workflow of the experiment. NHEKs were treated with control or 5 μM difamilast for 24 h. The supernatant of the NHEKs was collected. KU816 cells were cultured in the supernatant of NHEKs and stimulated with 10 ng/mL of IL-33 for 10, 30, or 60 min. (**B**) Western blotting. Phosphorylation of IL-33 downstream signaling proteins in KU816 cells was analyzed. Representative images from triplicate experiments with similar results are shown. (**C**) qRT-PCR analysis. KU812 cells cultured in the supernatant of control- or difamilast-treated NHEKs were stimulated with 10 ng/mL of IL-33 for 1 h or 3 h and mRNA expression was analyzed. Data are expressed as mean ± S.D.; N = 3/group. * *p* < 0.05; Tukey’s multiple comparison test.

We further investigated whether the sST2 produced in the supernatant by difamilast treatment could suppress the activity of IL-33. We treated NHEKs with either 5 μM difamilast or control for 24 h and then collected the supernatants. We then stimulated KU812 cells, a human basophil cell line, with the supernatant and IL-33 (10 ng/mL) (Figure 2A). Basophils are one of the direct target leukocytes for IL-33 in allergic inflammation and Th2 polarization such as AD [22]. KU812 cells are reported to express ST2L and IL-33 activates both NF-κB and MAPK pathways [23]. Stimulation with IL-33 for 10, 30, and 60 min was used to evaluate NF-κB and MAPK phosphorylation by western blotting (Figure 2B). The data on which the densitometric analysis is based are shown in Supplementary Figure S1. As shown in Figure 2B, the supernatant from difamilast-treated NHEKs inhibited IL-33-induced NF-κB and JNK phosphorylation in KU812 cells. IL-33 stimulation did not induce p38 and ERK phosphorylation in KU812 cells, which is partially consistent with a previous report [23]. IL-33 has been reported to upregulate TNF-α, IL-5, IL-13, and IL-4 in human basophils [22,23,24]. We observed that the supernatant from difamilast-treated NHEKs inhibited the upregulation of TNF-α induced by IL-33 stimulation for 1 h and the upregulation of IL-5 and IL-13 induced by IL-33 stimulation for 3 h (Figure 2C). These results suggest that difamilast treatment may inhibit IL-33 activity via sST2 production by NHEKs.

### 2.3. Difamilast Treatment Inhibited IL-33 Activity via sST2 Production by NHEKs

**Figure 3:**
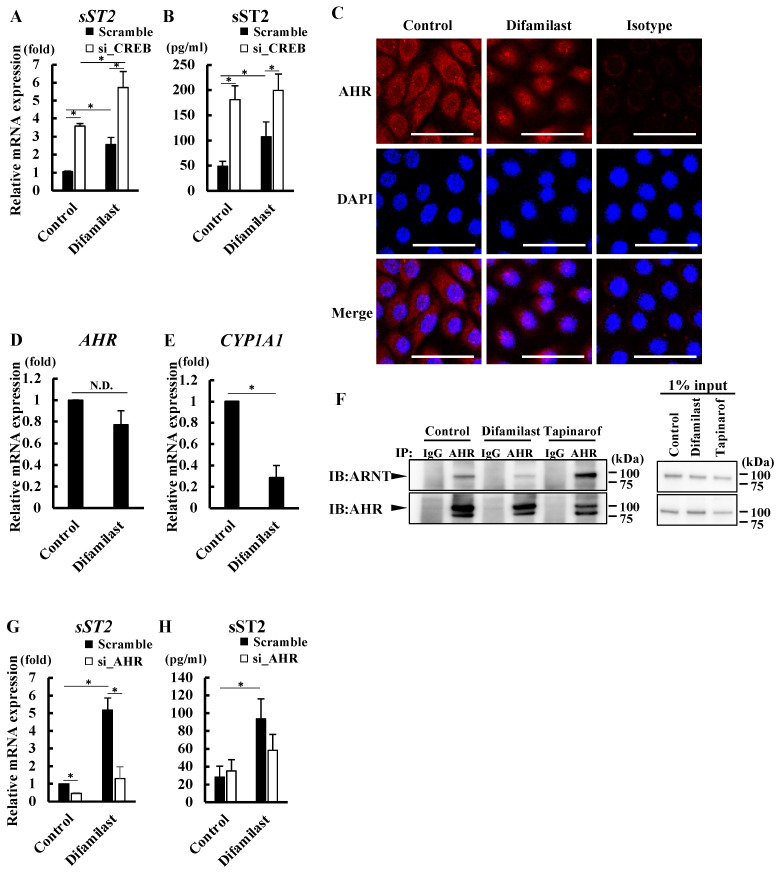
Difamilast treatment produced sST2 via aryl hydrocarbon receptor (AHR) in NHEKs. (**A**,**B**,**G**,**H**) NHEKs were transfected with scrambled (Scramble) siRNA, siRNA against CREB (si_CREB) (**A**,**B**), or siRNA against AHR (si_AHR) (**G**,**H**) and then treated with difamilast (5 μM) for 24 h. (**C**–**E**) NHEKs were treated with difamilast (5 μM) for 3 h. (**A**,**D**,**E**,**G**) qRT-PCR. (**B**,**H**) ELISA on supernatant. Data are expressed as mean ± S.D.; N = 3/group. * *p* < 0.05; Tukey’s multiple comparison test (**A**,**B**,**G**,**H**) or Student’s unpaired two-tailed *t*-test (**D**,**E**). (**C**) Immunohistochemical analysis. NHEKs were treated with 5 μM difamilast or control for 24 h. AHR (red) expression is shown with nuclear staining (blue, DAPI). Bar = 50 μm. Images are representative of three independent experiments. (**F**) Co-IP analysis. NHEKs were treated with 5 μM difamilast or 0.5 μM tapinarof or control for 1 h. The cell extract reacted with anti-AHR antibody. ARNT expression among precipitated proteins was analyzed by immunoblotting. Representative images from three separate experiments are shown.

We further investigated the mechanism by which difamilast treatment induces sST2 production in NHEKs. Since we previously showed that PDE4 inhibition by difamilast treatment induces CREB phosphorylation [5], we analyzed difamilast-treated NHEKs transfected with siRNA of CREB. Although CREB was successfully knocked down by siRNA transfection (Supplementary Figure S2A), this knockdown did not reverse the increases of sST2 mRNA and protein levels induced by difamilast treatment (5 μM) for 24 h (Figure 3A,B).

Since PDE4 inhibition by roflumilast and cAMP stimulation reportedly induced the nuclear translocation of aryl hydrocarbon receptor (AHR), a ligand-activated transcription factor [25,26], we examined whether difamilast treatment affected the intracellular localization of AHR in NHEKs. We observed the cytoplasmic expression of AHR in control-treated NHEKs and the nuclear expression of AHR in difamilast (5 μM)-treated NHEKs for 24 h, indicating that difamilast treatment induces the nuclear translocation of AHR in NHEKs (Figure 3C). We also confirmed that difamilast treatment did not alter the mRNA levels of *AHR* (Figure 3D). To further investigate the effect of difamilast treatment on AHR signaling, we measured mRNA levels of *CYP1A1*, a classical AHR downstream gene [27], in difamilast (5 μM)-treated NHEKs for 24 h. Consistent with a previous report [25], difamilast treatment downregulated rather than upregulated *CYP1A1* (Figure 3E). Because it has been reported that AHR and AHR nuclear translocator (ARNT) do not form a complex when AHR is driven to the nucleus by the elevation of cAMP [25,26], we examined whether ARNT coprecipitates with AHR in difamilast-treated NHEKs. We treated NHEKs with 5 μM difamilast or 0.5 µM tapinarof, an AHR modulator [27], or control for 1 h. We performed a co-immunoprecipitation (Co-IP) assay to pull down the AHR–ARNT complexes. As shown in Figure 3F, ARNT did not coprecipitate with AHR in difamilast-treated NHEKs, but it did in tapinarof-treated ones. We speculated that the reason why cAMP-induced activation of AHR signaling does not increase CYP1A1 expression is that AHR translocated into the nucleus by cAMP does not form a complex with ARNT, which is required for CYP1A1 expression [28,29].

Since we found that difamilast induced the nuclear translocation of AHR, we examined whether AHR was involved in the mechanism of sST2 production in difamilast-treated NHEKs. We transfected NHEKs with siRNA of AHR and then treated them with difamilast (5 μM) for 24 h. Knockdown of AHR was confirmed by qRT-PCR and western blotting (Supplementary Figure S2B). We observed that knockdown of AHR by siRNA transfection abolished the increases in mRNA levels of *sST2* induced by difamilast treatment (Figure 3G) and is likely to attenuate the increases in protein levels of sST2 induced by difamilast treatment (Figure 3H), suggesting that difamilast treatment produced sST2 via AHR in NHEKs.

### 2.4. Difamilast Treatment Produced sST2 via the AHR–NRF2 Axis in NHEKs

**Figure 4:**
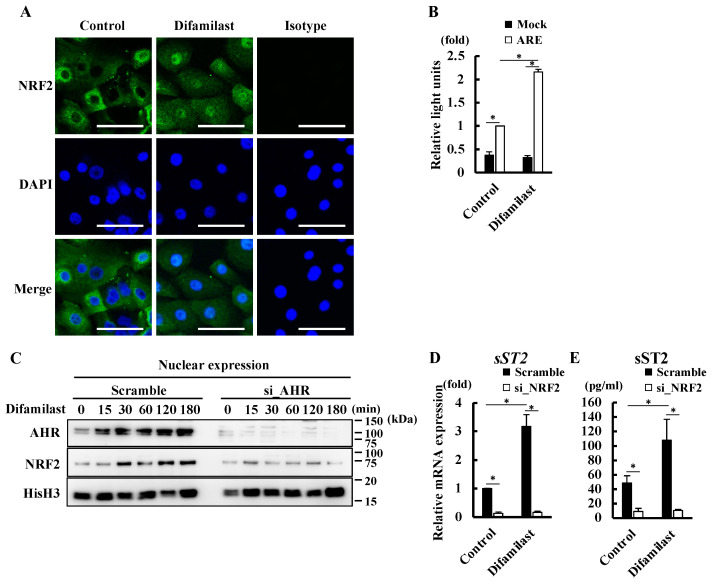
Difamilast treatment produced sST2 via the AHR–NRF2 axis in NHEKs. (**A**) Immunohistochemical analysis. NHEKs were treated with 5 μM difamilast or control for 24 h. NRF2 (green) expression is shown with nuclear staining (blue, DAPI). Bar = 50 μm. Images are representative of three independent experiments. (**B**) NHEKs were transfected with ARE-luciferase vector and treated with 5 μM difamilast or control for 3 h. Luciferase activity is shown as fold change, with that of mock-transfected control-treated cells as 1. Data are expressed as mean ± S.D.; N = 3/group. * *p* < 0.05; Tukey’s multiple comparison test. (**C**) Western blot analysis of nuclear expression of AHR and NRF2. NHEKs transfected with scrambled siRNA (Scramble) or AHR siRNA (si_AHR) were treated with 5 μM difamilast for 15, 30, 60, 120, or 180 min. Nuclear proteins were extracted. Representative images from three separate experiments are shown. (**D**,**E**) NHEKs were transfected with scrambled siRNA (Scramble) or siRNA against NRF2 (si_NRF2) and then treated with difamilast (5 μM) for 24 h. (**D**) qRT-PCR. (**E**) ELISA on supernatant. (**D**,**E**) Data are expressed as mean ± S.D.; N = 3/group. * *p* < 0.05; Tukey’s multiple comparison test.

We previously showed that AHR is a sensor of nuclear factor erythroid 2-related factor 2 (NRF2) activation, which is a master switch of cytoprotective gene expression [30]. To determine whether NRF2 was also involved in the mechanism by which difamilast treatment produced sST2 via AHR, we examined whether difamilast treatment affected the nuclear translocation of NRF2 using immunohistochemical staining analysis. We observed the cytoplasmic expression of NRF2 in control-treated NHEKs and the nuclear expression of NRF2 in difamilast-treated (5 μM) NHEKs for 24 h (Figure 4A), indicating that difamilast induces the nuclear translocation of NRF2 in addition to AHR. We also confirmed that difamilast (5 μM) treatment for 6 h increased the activity of antioxidant responsive element (ARE) (Figure 4B), an NRF2 binding site [30], in NHEKs transfected with ARE-luciferase vector, indicating that difamilast treatment activates NRF2. Since NRF2 activation is also induced by the production of reactive oxygen species (ROS) [30], we examined whether difamilast treatment (5 μM) for 24 h affected ROS production in NHEKs. Difamilast treatment did not increase ROS production compared with that in the control. BaP was used as a positive control for ROS production in NHEKs [30] (Supplementary Figure S3).

To clarify whether AHR is involved in the nuclear translocation of NRF2 induced by difamilast treatment, we measured the nuclear expression of NRF2 in difamilast-treated NHEKs transfected with siRNA of AHR. Difamilast treatment increased the nuclear expression of AHR at 15 min and the nuclear expression of NRF2 at 30 min. Furthermore, transfection of AHR siRNA abolished the difamilast-induced nuclear expression of AHR and NRF2 (Figure 4C), suggesting that difamilast treatment activates the AHR–NRF2 axis in NHEKs.

We next examined the involvement of NRF2 in the mechanism by which difamilast treatment produced sST2. Knockdown of NRF2 abrogated the increases in sST2 mRNA and protein levels induced by difamilast (5 μM) for 24 h (Figure 4D,E), indicating that difamilast treatment also produced sST2 via NRF2. The knockdown of NRF2 was confirmed via PCR and western blotting (Supplementary Figure S2). These findings suggest that difamilast treatment produces sST2 via the AHR–NRF2 axis in NHEKs.

## 3. Discussion

This study showed that difamilast treatment produced sST2 in NHEKs. It has been reported that sST2 is expressed in the epidermis of normal skin and its expression is further increased in Behçet’s disease [31]. However, few reported studies have examined sST2 expression in human keratinocytes in in vitro models. Primary cultures of human keratinocytes, melanocytes, and fibroblasts have low levels of sST2 expression in the absence of stimulation [32]. Although the mechanism of sST2 production in epithelial cells remains largely unknown, murine squamous cell carcinoma cells with nuclear expression of focal adhesion kinase (FAK), a kinase involved in tumor cell growth, have been reported to produce sST2, suggesting that FAK is associated with the transcription factors that regulate sST2 expression [33]. In addition, sST2 production is induced by IL-1a, IL-1b, and TNF-a stimulation and inhibited by NF-κB inhibitor in alveolar epithelial cells, suggesting that NF-κB activation is involved in the above-mentioned mechanism [32]. We found that the knockdown of NRF2 significantly suppressed the production of sST2 in NHEKs, clarifying the involvement of NRF2 in the mechanism of sST2 production. Since difamilast treatment did not increase ROS production, we believe that NRF2 was activated via AHR without oxidative stress. It has also been reported that IL-1a, IL-1b, and TNF-a produce ROS [34,35], which triggers the nuclear translocation of NRF2 [36], resulting in the production of sST2.

Difamilast treatment produced sST2 but had no effect on ST2L expression at the mRNA and protein levels. There are two promoters in the ST2 gene, a proximal promoter and a distal one, both of which affect sST2 and ST2L mRNA expression. The proximal promoter controls the expression of sST2, while the distal promoter is involved in the expression of both sST2 and ST2L [12]. It is thus possible that NRF2 translocated into the nucleus by difamilast treatment increased the expression of sST2 through the proximal promoter. Since difamilast treatment induces CREB phosphorylation [5], we knocked down CREB and found that this increased sST2 production. These results indicate that CREB has a suppressive effect on the transcriptional activity of sST2 expression, suggesting that sST2 production by NHEKs may be balanced by NRF2 and CREB.

**Figure 5:**
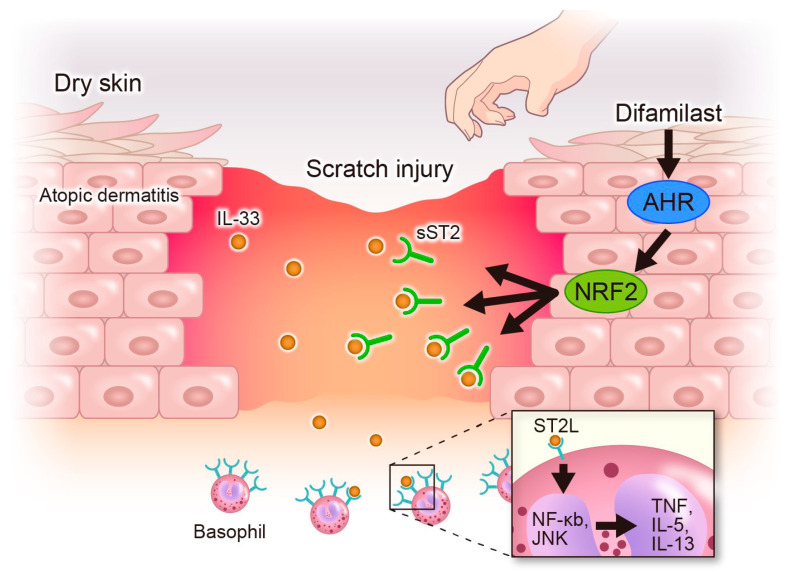
Difamilast treatment produces sST2 via the AHR–NRF2 axis, which inhibits IL-33-induced basophil activation. IL-33, a pruritic cytokine, is highly expressed in keratinocytes of AD and dry skin. IL-33 is secreted into the extracellular space via cell death such as necrosis induced by scratch injury. Secreted IL-33 activates basophils via ST2L binding and phosphorylation of NF-κB and JNK, leading to the upregulation of TNF-α, IL-5, and IL-13. Topical difamilast treatment produces sST2 via the AHR–NRF2 axis in keratinocytes. The sST2 blocks IL-33 binding to the ST2L receptor, resulting in inhibition of basophil activation.

We found that sST2 produced by difamilast-treated NHEKs inhibited the IL-33-induced phosphorylation of NF-κB and MAPK in KU812 cells. As mentioned above, basophils are one of the target immune cells activated by IL-33 [22] and regulate and induce chemotaxis of other inflammatory cells, further promoting the process of allergic skin inflammation [37]. Since IL-33 reportedly induces the upregulation of TNF-α, IL-5, IL-13, and IL-4 via NF-κB and MAPK activation [23], we measured the gene expression and found the inhibitory effect of difamilast on IL-33-induced upregulation of TNF-α, IL-5, and IL-13 (Figure 2C). TNF-α induces spongiosis in human skin equivalents, increases thymic stromal lymphopoietin secretion by keratinocytes, and alters the expression of early and terminal differentiation proteins, contributing to skin barrier dysfunction [38]. TNF-α also sensitizes nociceptive nerve endings via TNF-α receptors and increases their responsiveness [39], resulting in intense pruritus. IL-5 is one of the Th2 cytokines involved in the differentiation, maturation, migration, development, survival, trafficking, and effector function of eosinophils, in addition to basophils and mast cells. IL-5-producing Th2 lymphocytes are reportedly increased in the peripheral blood and AD lesions, resulting in the infiltration of eosinophils [40]. It has been shown that IL-33 promotes IL-5 production in KU812 cells, which is suppressed by sST2 administration [41]; this is consistent with our results. Basophils are an important source of IL-13 in pruritic skin diseases [42]. It has also been reported that IL-33 can modulate the activity of basophils in IgE-dependent and IgE-independent manners [43]. These findings suggest that the capacity of difamilast to inhibit IL-33-induced upregulation of TNF-α, IL-5, and IL-13 may contribute to improving the disease activity of AD. IL-33 has also been reported to be elevated in dry skin pruritus [19]. IL-33 causes pruritus via ST2L on sensory neurons in animal models of dry skin. In addition, IL-33 enhances histaminergic pruritus in sensory neurons [19]. A possible mechanism for this is that IL-33 promotes pruritus via a pathway involving IL-13 production, which may enhance histamine-induced pruritus [19]. These findings suggest that difamilast treatment may be effective against pruritus in AD and dry skin by interfering with IL-33 activity (Figure 5).

This study is limited by the fact that the experimental results were obtained in vitro, so further analyses in animal models of AD and clinical specimens from patients with AD are required. It has been suggested that sST2 enhances IL-33 activity through the formation of the sST2–IL-33 complex in vivo [44]. It is thus possible that the suppression of IL-33 signaling by sST2 adversely affects the pathogenesis of AD, but further studies of this issue are needed. In conclusion, this study revealed a novel mechanism of difamilast, which supports its clinical efficacy in the treatment of AD.

## 4. Materials and Methods

### 4.1. Materials

Difamilast and tapinarof, purchased from MedChemExpress (Middlesex, NJ, USA), were dissolved in DMSO and stored at −80 °C. Benzo[a]pyrene (BaP) was purchased from Sigma-Aldrich (St. Louis, MI, USA) and dissolved in DMSO.

### 4.2. Cell Culture

NHEKs were purchased from Lonza (Basel, Switzerland) and cultured in KGM-gold (Lonza) supplemented with SingleQuots (Lonza). The NHEKs were obtained from the epidermis of adult skin from a single donor. KU812 (RCB0495) cells were provided by the RIKEN BRC through the National BioResource Project of the MEXT/AMED, Japan. KU812 is a human basophil cell line that was established from the peripheral blood of a 38-year-old male, Asian patient with chronic myelogenous leukemia. The cells were maintained in RPMI supplemented with 10% inactivated fetal bovine serum and antibiotics.

### 4.3. Quantitative Real-Time Polymerase Chain Reaction (qRT-PCR) Analysis

Total RNA was isolated using the RNeasy mini kit (Qiagen, Venlo, The Netherlands) and reverse-transcribed to cDNA using the SuperScript RT reagent kit (Takara Bio, Otsu, Japan). qRT-PCR was performed using a CFX connect real-time PCR system (Bio-Rad Laboratories, Hercules, CA, USA) with TB Green Premix Ex Taq (Takara Bio) or TaqMan Fast Advanced Master Mix (Thermo Fisher Scientific, Waltham, MA, USA). Primers and TaqMan probes are listed in Supplementary Table S1. Relative mRNA expression was normalized with *ACTB* or *YWHAZ*.

### 4.4. Enzyme-Linked Immunosorbent Assay (ELISA)

The concentration of sST2 in the supernatant was measured in accordance with the manufacturer’s instructions (R&D Systems, Minneapolis, MN, USA). Briefly, 96-well plates were coated with capture antibody in phosphate-buffered saline overnight. After blocking with reagent diluent buffer, the microplates were incubated with frozen supernatant or standard for 2 h and reacted with detection antibody for 2 h. Binding was detected with streptavidin-horseradish peroxidase. The enzymatic reaction was stopped with stopping buffer, and the absorbance was measured at 450 nm.

### 4.5. Stimulation of KU812 Cells

NHEKs cultured in epidermal growth factor- and hydrocortisone-free medium were incubated with either 5 μM difamilast or vehicle control (DMSO) for 24 h. KU812 cells were stimulated with the supernatant and IL-33 (10 ng/mL) for the indicated times (Figure 2A).

### 4.6. Small Interfering RNA (siRNA) Transfection

siRNA targeting AHR (s1200), NRF2 (s9492), and CREB (s3490) and scrambled siRNA (Silence Negative Control No. 1) were purchased from Thermo Fisher Scientific. Lipofectamine RNAiMAX Transfection Reagent (Thermo Fisher Scientific) was used for siRNA transfection.

### 4.7. Protein Extraction

Cells were lysed with RIPA lysis buffer (50 mM Tris-HCl at pH 7.4, 150 mM sodium chloride, 5 mM EDTA, 0.1% SDS, 1% NP40, 1% deoxycholate, 10 mM NaF) supplemented with protease inhibitor cocktail (Sigma-Aldrich) and PhosSTOP (Roche Diagnostics, Rotkreuz, Switzerland). NE-PER nuclear and cytoplasmic extraction reagents (Thermo Fisher Scientific) were used for nuclear protein extraction.

### 4.8. Western Blotting

The extracted proteins were denatured with LDS sample buffer (Thermo Fisher Scientific) containing DTT (Thermo Fisher Scientific). Equal amounts of protein were loaded onto a 4–12% SDS-PAGE gel (Thermo Fisher Scientific) and blotted onto a PVDF membrane (Merck Millipore, Burlington, MA, USA). Antibodies against ST2L (PA5-23316; Thermo Fisher Scientific), IL-33 (Nessy-1; Abcam, Cambridge, UK), NRF2 (EP1808Y; Abcam), histone H3 (1G1; Santa Cruz Biotechnology, Dallas, TX, USA), AHR (D5S6H), ARNT (D28F3), β-Actin (8H10D10), p38 MAPK (D13E1), NF-κB (D14E12), JNK (#9252S), ERK (#9102), phospho-p38 MAPK (Thr180/Tyr182; D3F9), phospho-JNK (Thr183/Tyr185; 81E11), phospho-NF-κB p65 (Ser536; 93H1), and phospho-ERK (D13.14.4E; Cell Signaling Technology, Danvers, MA, USA) were used. Peroxidase-conjugated secondary antibodies (Cell Signaling Technology) were reacted with SuperSignal West Pico (Thermo Fisher Scientific) and detected using a ChemDoc imaging system (Bio-Rad Laboratories). Densitometric analysis was performed using Image Lab 5.2 (Bio-Rad Laboratories).

### 4.9. Luciferase Assay for Antioxidant Response Element (ARE) Activity

NHEKs were transfected with pGL4.37-ARE luc plasmid with control vector pRL-CMV (both from Promega, Madison, WI, USA) using X-tremeGENE HP DNA transfection reagent (Sigma-Aldrich). Transfected cells were incubated with difamilast or control vehicle for 24 h, and luciferase activity was measured using the dual-luciferase receptor assay system (Promega).

### 4.10. Analysis of Reactive Oxygen Species (ROS) Production

NHEKs cultured on black glass-bottomed plates were treated with 5 μM difamilast or control vehicle with or without 1 μM BaP for 24 h. ROS production was visualized using photooxidation-resistant DCFH-DA (Dojindo, Kumamoto, Japan) and observed with an EVOS cell imaging system (Thermo Fisher Scientific).

### 4.11. Immunohistochemical Analysis

Acetone-fixed NHEKs were used for AHR staining. PFA-fixed cells (4%) were used for NRF2 staining. Fixed cells were permeabilized with 0.1% Triton-X100 for 10 min and then stained with anti-AHR antibody (1:250) or anti-NRF2 antibody (1:250). Alexa Fluor 546 anti-rabbit IgG or Alexa Fluor 488 anti-rabbit IgG (both from Thermo Fisher Scientific) was then used for secondary staining. Cells were mounted with UltraCruz mounting medium containing 4′,6-diamidino-2-phenylindole (DAPI) (Santa Cruz Biotechnology) for nuclear staining and analyzed using a TCS SP8 confocal laser scanning microscope (Leica, Wetzlar, Germany).

### 4.12. Co-IP Assay

NHEKs were treated with 5 μM difamilast or 0.5 μM tapinarof or control vehicle (DMSO) for 1 h, and then co-IP assay was performed using Pierce Classic IP Kit (Thermo Fisher Scientific). Whole-cell extract was precleared with control agarose resin for 1 h and then incubated with anti-AHR antibody or control rabbit IgG for 1 h with gentle mixing. Immune complexes were resolved using sodium dodecyl sulfate sample buffer, and ARNT was detected by western blotting with anti-ARNT antibody.

### 4.13. Statistical Analysis

Statistical significance of differences between groups was determined using Student’s unpaired two-tailed *t*-test (for two groups), Dunnett’s multiple comparison test (for multiple groups compared to control), or Tukey’s multiple comparison test (for multiple groups) using JMP software (JMP, Cary, NC, USA). A *p*-value less than 0.05 was considered significant.

## Data Availability

The authors confirm that the data supporting the findings of this study are available within the article and its Supplementary Materials.

## Supplementary Materials

The following supporting information can be downloaded at: https://www.mdpi.com/article/10.3390/ijms25147910/s1.

## Abbreviations

AD: Atopic dermatitis; AHR: Aryl hydrocarbon receptor; cAMP: Cyclic adenosine monophosphate; CREB: cAMP response element binding protein; IL: Interleukin; MAPK: Mitogen-activated protein kinase; NF-κB: Nuclear factor-kappa B; NRF2: Nuclear factor erythroid 2-related factor 2; PDE: Phosphodiesterase; ST2: suppression of tumorigenicity 2 protein; TNF: Tumor necrosis factor.
