# Supplementary material for: Difamilast, a Topical Phosphodiesterase 4 Inhibitor, Produces Soluble ST2 via the AHR–NRF2 Axis in Human Keratinocytes
Source: Int J Mol Sci. 2024 Jul 19;25(14):7910. doi: 10.3390/ijms25147910 (PMC11277015; doi:10.3390/ijms25147910)
Supplement: Supplementary file 1 [file ijms-25-07910-s001.zip › ijms-3053258-supplementary.pdf]

Supplementary Figure S1

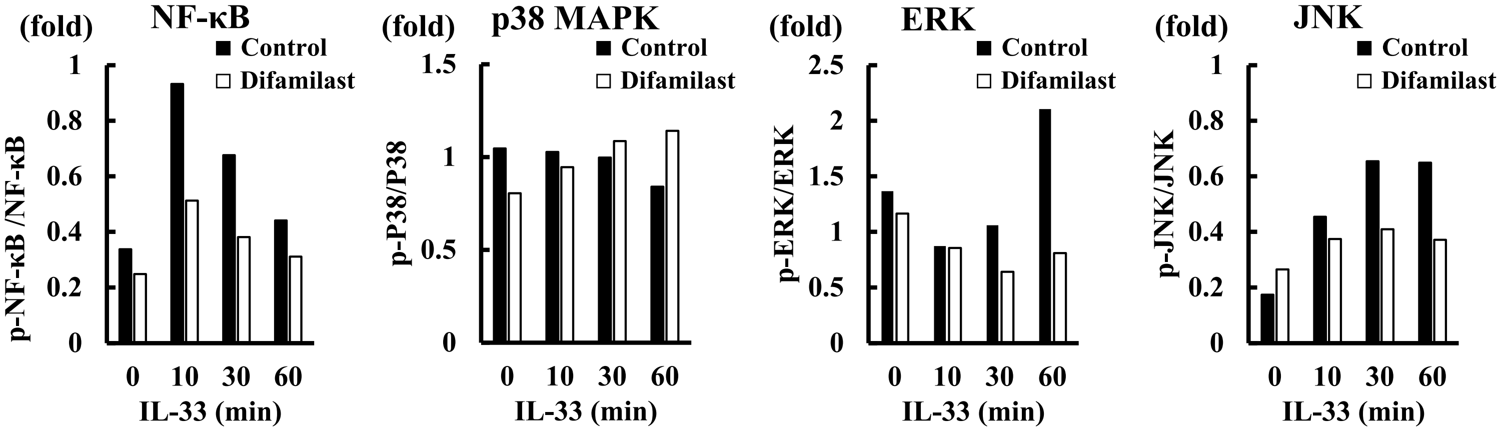

Densitometric analysis of NF-κB and MAPK phosphorylation. NHEKs were treated with control or 5 μM difamilast for 24 h. The supernatant of the NHEKs was collected. KU816 cells were cultured in the supernatant of NHEKs and stimulated with 10 ng/mL IL-33 for 10, 30, or 60 min. Phosphorylation of IL-33 downstream signaling proteins in KU816 cells was analyzed by western blotting. The densitometric analysis of the data was performed using Image Lab **5.2**

Supplementary Figure S2

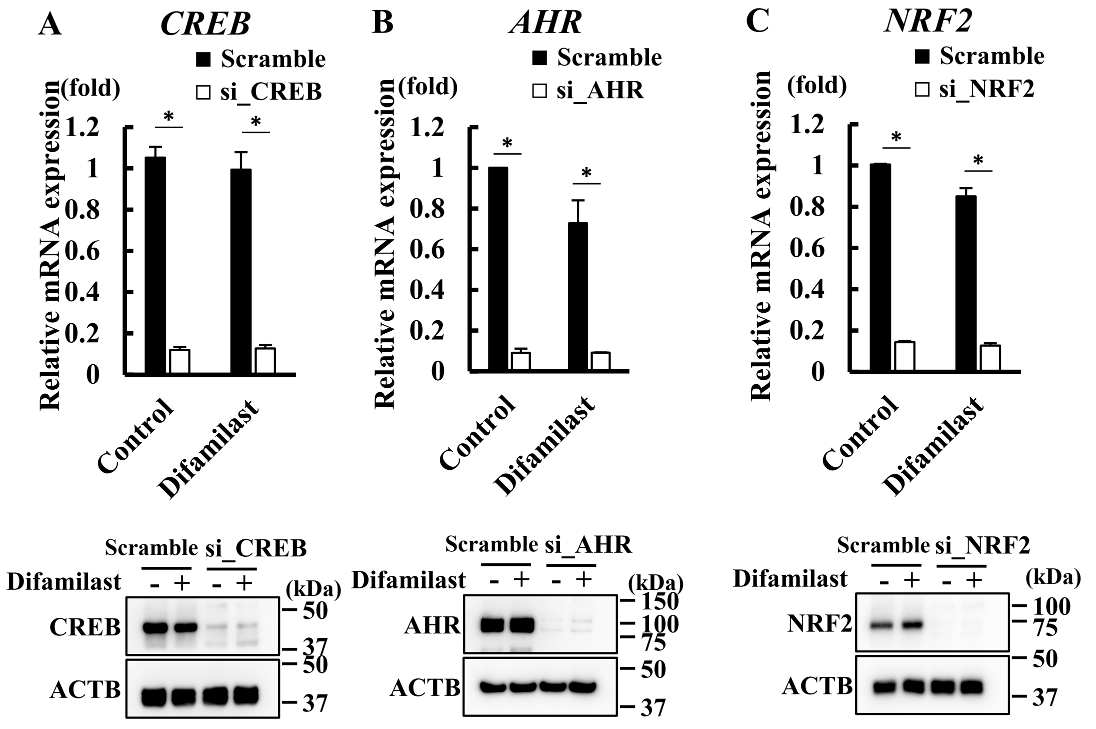

Knockdown efficiency by siRNA transfection.
The efficiency of siRNA transfection against CREB, AHR, and NRF2 was analyzed by qRT-PCR and western blot analysis. (A–C) Data are expressed as mean ± S.D.; N = 3/group. *$p < 0.05$; Tukey's multiple comparison test. Western blot images are representative of three separate experiments.

Supplementary Figure S3

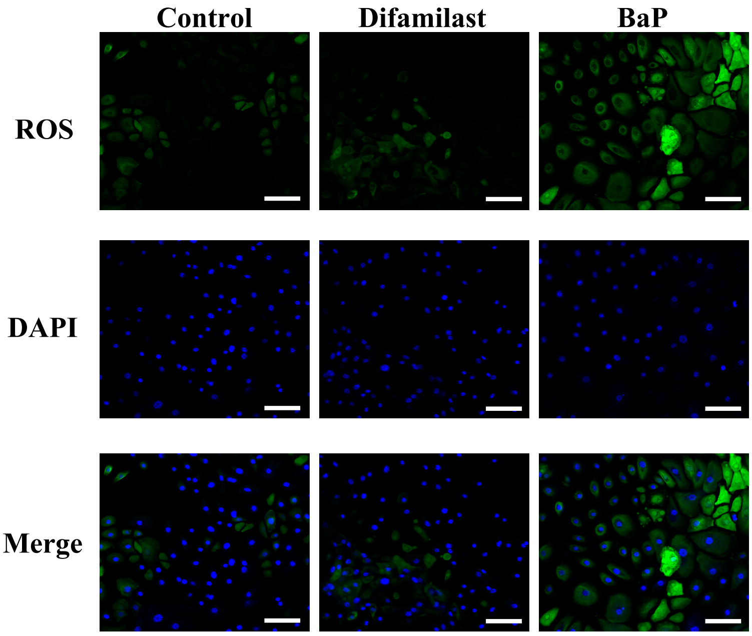

Difamilast treatment did not increase ROS production.
NHEKs were treated with 5 μM difamilast or control, or 1 μM benzo[a]pyrene (BaP) for 24 h. ROS production was visualized with photooxidation-resistant DCFH-DA (green) with counterstaining of nuclei (DAPI: blue). Representative images from three separate experiments are shown. Bar = 50 μm.

Supplementary Table S1 List of primers and probe

Gene symbol	Forward primer 5'－3'	Reverse primer 5'－3'
YWHAZ	tgaggttgccgctggtgatg	cagtctgataggatgtgttggttgc
AHR	caaatccttccaagcggcata	cgctgagcctaagaactgaaag
CYP1A1	tagacactgatctggctgcag	gggaaggctccatcagcatc
IL-33	agccttgtgtttcaagctggg	ttgtgctttctacctgttttcagtg
NRF2	tcagcgacggaaagagtatga	ccactggtttctgactggatgt
CREB	ctgggagaagcggagtgttg	atgtggcaatctgtggctgg
TNF-α	ccaggcagtcagatcatcttctc	agcttgagggtttgctacaacat
IL-5	tggagctgcctacgtgtatg	ttcgatgagtagaaagcagtgc
IL-13	cctcatggcgcttttgttgac	tctggttctgggtgatgttga
IL-4	ccaactgcttccccctctg	tctgttacggtcaactcggtg

Gene symbol	Taqman probe
YWHAZ	Hs01122445_g1
sST2	Hs01073297_m1
ST2L	Hs00249389_m1
